# Identification of target genes for spermatogenic cell-specific KRAB transcription factor ZFP819 in a male germ cell line

**DOI:** 10.1186/s13578-016-0132-4

**Published:** 2017-01-03

**Authors:** Sora Jin, Heejin Choi, Jun Tae Kwon, Jihye Kim, Juri Jeong, Jaehwan Kim, Seong Hyeon Hong, Chunghee Cho

**Affiliations:** School of Life Sciences, Gwangju Institute of Science and Technology, Gwangju, 61005 South Korea

**Keywords:** Apoptosis, Chromatin-immunoprecipitation, Germ cell, KRAB, Microarray, Spermatogenesis, Testis

## Abstract

**Background:**

*Zfp819*, a member of the *Krüppel*-associated box (KRAB) family, encodes a spermatogenic cell-specific transcription factor. *Zfp819*-overexpression induces apoptosis and inhibits proliferation in somatic cell lines.

**Results:**

In the present study, we examined the cellular effects of *Zfp819* in a male germ cell line (GC-2 cells). Overexpression of *Zfp819* demonstrated an increase in the number of apoptotic cells, leading to inhibition of proliferation in GC-2 cells. We further investigated genes regulated by ZFP819 using microarray analysis and chromatin-immunoprecipitation combined with microarray analysis (ChIP-chip) in GC-2 cells. We identified 118 downregulated genes in *Zfp819*-overexpressing GC-2 cells using microarray analysis. ChIP-chip assay revealed that 1011 promoter sites (corresponding to 262 genes) were specifically enriched in GC-2 cells transfected with *Zfp819*. Two genes (trinucleotide repeat containing 6b and annexin A11) were commonly found when we compared the data between microarray and ChIP-chip analyses. Consistent with these results, *Zfp819* overexpression significantly reduced the transcript levels of the two genes by binding to their promoter regions. Tissue distribution analysis indicated that both genes were predominantly expressed in testis. It has been reported that these two genes function in apoptosis.

**Conclusion:**

Collectively, our study provides inclusive information on germ cell-specific gene regulation by ZFP819, which is involved in apoptosis, to maintain the integrity of spermatogenesis.

**Electronic supplementary material:**

The online version of this article (doi:10.1186/s13578-016-0132-4) contains supplementary material, which is available to authorized users.

## Background

Spermatogenesis is a unique and highly elaborated process that requires a highly organized and tightly regulated network of genes, many of which are spermatogenic-cell-specific. Previous reports investigated gene expression profiles in mouse spermatocytes and round spermatids and found that the proportions of testis-specific genes in spermatocytes and round spermatids were 11% (230 genes) and 22% (467 genes), respectively [1, 2]. Despite the presence of many unique genes expressed in spermatogenic cells, the characteristics of their associated transcriptional network(s) are largely unknown.

The *Krüppel*-associated box and C2H2-type zinc finger motifs (KRAB-ZF) family, one of the largest transcription factor groups in vertebrates has a transcriptional repressive activity by binding to DNA in a sequence-specific manner [3]. The members of KRAB-ZF family are generally expressed in various tissues and are involved in diverse processes including apoptosis, cell proliferation, and tumorigenesis [3]. It has been reported that a number of the KRAB-ZF genes on a certain chromosome are abundantly expressed in testis and fetal brain and may have important functions in these tissues [4]. Previously, we comprehensively identified and characterized the KRAB-ZF genes in the reproductive tissues (testis or ovary) [5]. Three KRAB-ZF genes were specifically or predominantly expressed in the gonads. Of them, *Zfp819*, which was specific to spermatogenic cells, was analyzed in detail. We found that the overexpression of *Zfp819* affected cell proliferation and induced apoptosis in somatic cell lines. The overexpression of *Zfp819* changed the expression of B cell lymphoma protein-2 (BCL-2) and poly(ADP-ribose) polymerase (PARP) [5].

To date, the target genes of only a few KRAB-ZF members have been discovered [6–9]. In the present study, we investigated the transcriptional network of *Zfp819* through genome-wide approaches in a germ cell line. We found that the overexpression of *Zfp819* affected cell proliferation and induced apoptosis in GC-2 cells. Microarray analysis revealed 1737 differentially expressed genes in *Zfp819*-overexpressing cells compared with mock-transfected cells, and of them, 118 genes were down-regulated by ZFP819. In addition, chromatin immunoprecipitation (ChIP)-chip analysis revealed 1011 promoter sites enriched by ZFP819. Interestingly, two genes encoding trinucleotide repeat containing 6b (TNRC6B) and annexin A11 (ANXA11) were found to overlap between the microarray and ChIP results. These genes were verified by ChIP-PCR and promoter assays. They also demonstrated a predominant expression in testis and were previously reported to have important functions in apoptosis. Collectively, our results revealed for the first time to the best of our knowledge using GC-2 cells a germ cell-specific gene regulation by ZFP819 that is involved in apoptosis.

## Methods

### RT-PCR and qRT-PCR

Mouse adult tissues and GC-2 cells transfected with pcDNA3.1/myc or pcDNA3.1/myc-*Zfp819* were assessed. All animal investigations were conducted according to the guidelines of the Animal Care and Use of the Gwangju Institute of Science and Technology. Total RNA samples were extracted using TRIzol™ Reagent (MRC) according to manufacturer’s protocol, and reverse transcribed with Omniscript reverse transcriptase (Qiagen). Complementary DNA samples prepared from mouse adult tissues were amplified with primers specific for each of the reproductive KRAB-ZF genes (Additional file 1: Table S1). Quantitative real-time PCR (qRT-PCR) was performed using SYBR Green *Taq* polymerase mix (TaKaRa Bio, Inc.). All of the reactions contained 10 µl of SYBR Green Master Mix and 50–100 ng of template cDNA. Glyceraldehyde 3-phosphate dehydrogenase *(Gapdh)* was used as an internal control.

### Dual-luciferase reporter assay

GC-2 cells (4.0 × 10^5^ cells/well) were seeded onto 24-well plates and incubated at 37 °C for 24 h. For repressive activity of *Zfp819*-KRAB, when the cells reached approximately 80–90% confluence, they were co-transfected with 250 ng of pcDNA3.1/myc-*Zfp819*-KRAB, 250 ng of a firefly luciferase-encoding vector (pGL3-promoter, Invitrogen), and 5 ng of pRL-TK (Renilla used as an internal control for normalization of transfection efficiency in the absence and presence of *Zfp819* overexpression). After 24 h, the cells were lysed with passive lysis buffer (Promega), dual luciferase assays were performed with a Luciferase Reporter Assay kit (Promega), and the luciferase activity was measured using a Centro LB 960 DLReady microplate illuminometer (Berthold Technologies). GAL4-DBD was used as a basic control, and KOX1-DBD was used as a positive control. For promoter activity of Zfp819, Tnrc6b or Anxa11 promoter regions were inserted into pGL-promoter (Invitrogen). When the cells reached approximately 80–90% confluence, they were co-transfected with 250 ng of pcDNA3.1/myc-*Zfp819* or pcDNA3.1/myc, 250 ng of a firefly luciferase-encoding vector (pGL3-promoter, Invitrogen), and 5 ng of pRL-TK (Renilla). Each experiment was repeated three independent times in triplicate.

### Cell proliferation assay using MTT

Cell proliferation was measured using the 3-(4,5-dimethylthiazol-2-yl)-2,5-diphenyltetrazolium bromide (MTT) assay. In brief, GC-2 cells were grown in 6-well plates for 1 day at a density of 3.0 × 10^5^cells/well. After an additional 24 h, cells were transfected with pcDNA3.1/myc or pcDNA3.1/myc-*Zfp819* plasmids using Lipofectamine 2000 (Invitrogen; 10 µl reagent per 5 µg DNA). After 48 h, the cells were exposed to 1 ml/well MTT solution (1.5 mg/ml) at 37 °C for 1.5 h in medium. The medium was removed, 0.04 N isopropanol in HCl was added to solubilize the formazan crystals, and the plates were gently agitated at room temperature for 10 min in the darkness. Cell proliferation was measured at 570 and 650 nm using an ELISA reader. Each experiment was performed three independent times in triplicate.

### Flow cytometry and TUNEL assay

Cells were transfected with pcDNA3.1/myc (empty vector) or pcDNA3.1/myc-*Zfp819* plasmids and incubated at 37 °C for 24 h. For the analysis of cell cycle distribution, GC-2 cells were seeded (3.0 × 10^5^ cells/well) in 6-well plates for 24 h, harvested with trypsin–EDTA (TE, Gibco), and fixed with 70% ethanol for 1.5 h on ice in the dark. The cells were then collected by centrifugation, washed once with ice-cold PBS, and incubated with 500 µl of propidium iodide (PI) solution (50 µg/ml) for 30 min at 37 °C in the dark. Finally, the cells were resuspended in PBS then analyzed by flow cytometry. For the detection of apoptotic cells, the TUNEL assay was performed with an Apop Tag® Plus Peroxidase In Situ Apoptosis Detection kit (Chemicon). Cells (3.0 × 10^5^ cells/well) were plated in 6-well plates, transfected as indicated for 48 h, and then fixed with 4% formaldehyde at room temperature for 10 min. The cells were then washed with PBS, mixed with 55 µl/well of TdT enzyme, and incubated at 37 °C in a humidified chamber for 1 h. The reaction was stopped with stop/wash buffer, DNA was counterstained with Hoechst 33,342 (Sigma), and the cells were visualized by confocal microscopy. Each experiment was performed three times in triplicate.

### Immunoblotting

The transfected cell lysates were prepared with 1% sodium dodecyl sulfate (SDS). Equal amounts of protein (30 µg) were separated by 8% SDS–polyacrylamide gel electrophoresis and transferred onto a polyvinylidene difluoride (PVDF) membranes (Millipore corporation). Membranes were hybridized for 17 h at 4 °C or 1 h at room temperature with primary antibodies including: anti-Myc (1:1000, Cell signaling), anti-TNRC6B (1:1000, Millipore), anti-ɑ-tubulin (1:1000, Millipore), and anti-GAPDH (1:1000, Bio-RAD) antibodies. Bound IgG was detected following 1 h incubation with horseradish peroxidase-conjugated secondary antibodies (Jackson ImmunoResearch Laboratories) and the Luminol and Stable peroxidase solutions (ThermoFisher scientific).

### Microarray analysis

GC-2 cells were transfected with pcDNA3.1/myc (empty vector) or pcDNA3.1/myc-*Zfp819* using Lipofectamine 2000 reagent (Invitrogen). Total RNA was extracted using RNeasy columns (Qiagen) according to the manufacturer’s instructions. For control and test RNAs, the synthesis of target cRNA probes and hybridization were performed using Agilent’s LowInput QuickAmp Labeling Kit (Agilent Technology, USA) as per the manufacturer’s instructions. The hybridized microarrays were washed as per the manufacturer’s washing protocol (Agilent Technology). This analysis was repeated three times.

### ChIP-chip assay

ChIP assay was performed using the Chromatin Immunoprecipitation Assay Kit (Millipore Upstate) as previously described [10]. In brief, GC-2 cells transfected with pcDNA3.1/myc (mock without protein expression) or pcDNA3.1/myc-*Zfp819* plasmids were cross-linked by adding 1% formaldehyde (37%) for 10 min in the humidified chamber (at 37 °C with 5% CO_2_), followed by a quenching step with 125 mM glycine. Cells were rinsed twice with 1× PBS and resuspended in 1% SDS lysis buffer containing protease inhibitor cocktail (PIC). Sonicated chromatin was immunoprecipitated with α-myc antibody (Cell Signaling). Antibody-bound chromatin complexes were then captured with protein A agarose beads blocked with salmon sperm DNA, and eluted in SDS buffer. Formaldehyde crosslinking was reversed, followed by DNA purification by phenol–chloroform extraction. The immunoprecipitated and input DNA were amplified using a whole genome amplification kit (GenomePlex® Complete Whole Genome Amplification Kit) as recommended by the manufacturer. Each 2.5–5 µg of cyanine 3-labeled and cyanine 5-labeled DNA target were mixed and then resuspended with 2× hybridization buffer, Cot-1 DNA, and Agilent 10× blocking agent. Before hybridization to the array, the 260 µl hybridization mixtures were denatured at 95 °C or 3 min and incubated at 37 °C for 30 min. The hybridization mixtures were centrifuged at 17,900×*g* for 1 min and directly pipetted onto the Mouse Promoter 2 × 400 K microarray. This array covers 415,814 probes (~19,000 genes as represented by RefSeq, probes are spaced 93 bp apart). The arrays hybridized at 65 °C for 40 h in an Agilent Hybridization oven (Agilent Technology). The hybridized microarrays were washed as per the manufacturer’s washing protocol (Agilent Technology).

### Gene silencing using siRNAs

siRNA-mediated gene silencing of Tnrc6b and Anxa11 was performed using siRNA duplexes (Bioneer). The target sequences of siRNAs were ACU UCU GGA GAC UAU CGA A(dTdT) (Anxa11) and CUG GUU ACC UGC CAA AUC U(dTdT) (Tnrc6b). In brief, GC-2 cells were grown in 6-well plates for 1 day at a density of 6.0 × 10^5^ cells/well. After an additional 24 h, cells were transfected with of control siRNA, Tnrc6b siRNA, or Anxa11 siRNA (25 nM) using Lipofectamine 2000 (Invitrogen; 6.25 µl reagent). After 48 h, total RNA was extracted using TRIzol™ Reagent (MRC) according to manufacturer’s protocol, and reverse transcribed with Omniscript reverse transcriptase (Qiagen). The knockdown efficiency was confirmed by RT-PCR with primers specific for *Tnrc6b* and *Anxa11*.

### Statistical analysis

Each experiment was performed three times. Each time, measurements were conducted on three samples with the same design and averaged. Data are represented as mean ± standard error of mean (mean ± SEM.). Statistical analysis was calculated by Student’s *t* test.

## Results

### Repressive activity of Zfp819 in germ cell line (GC-2 cell)

**Fig. 1:**
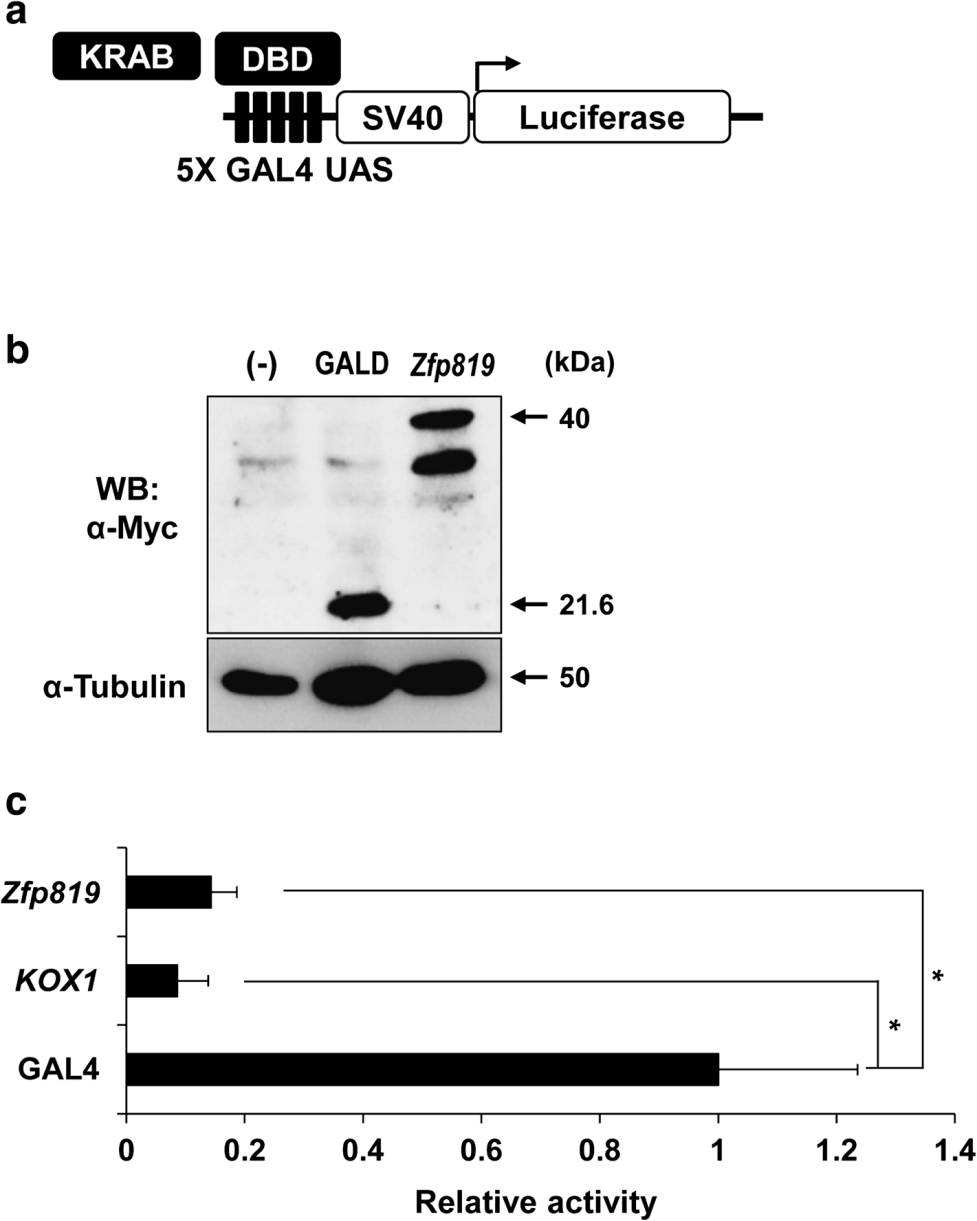
Transcriptional repression by the Zfp819 protein. **a** Diagram of the expression and reporter constructs used for the reporter assay. **b** Western blotting of GAL4-DBD-Myc and Zfp819-GAL4-DBD-Myc expressed in GC-2 cells. **c** GC-2 cells were transfected and subjected to reporter assays. Both KRAB-ZF genes had strong repressive activities. GAL4-DBD-Myc alone and KOX1 were used as controls. Experiments were conducted in triplicate. The data are expressed as the mean ± SEM; *p < 0.01 versus GAL4-DBD-Myc (Student’s *t* test)

We previously reported that the KRAB domain of *Zfp819* has repressive activity using the GAL4-UAS system in somatic cell lines (HEK 293T and NIH3T3) [5]. To confirm the repressive activity of *Zfp819* in the germ cell line as well, we performed a reporter assay using GAL4-DBD-Myc constructs fused to the KRAB domain sequences from *Zfp819* and a luciferase expression construct containing an enhancer that specifically bound to GAL4 (GAL4-UAS) (Fig. 1a). These two constructs were cotransfected into GC-2 cells, which are derived from mouse spermatocytes [11]. The controls included KOX1, which is a KRAB-ZF known to strongly repress transcription, and GAL4-DBD-Myc alone. Western blotting of cells transfected with a construct encoding Zfp819-GAL4-DBD-Myc confirmed that the recombinant protein was successfully expressed in GC-2 cells (Fig. 1b). Then, monitoring of luciferase activity demonstrated that each of the tested KRAB domains (*KOX1* and *Zfp819*) reduced luciferase activity compared with GAL4-DBD-Myc alone, indicating that these domains can repress transcription in GC-2 cells (Fig. 1c). Taken together, these results indicate that we successfully identified *Zfp819* with transcription-repressing activity in GC-2 cells.

### Inhibition of proliferation and induction of apoptosis in *Zfp819*-overexpressing cells

**Fig. 2:**
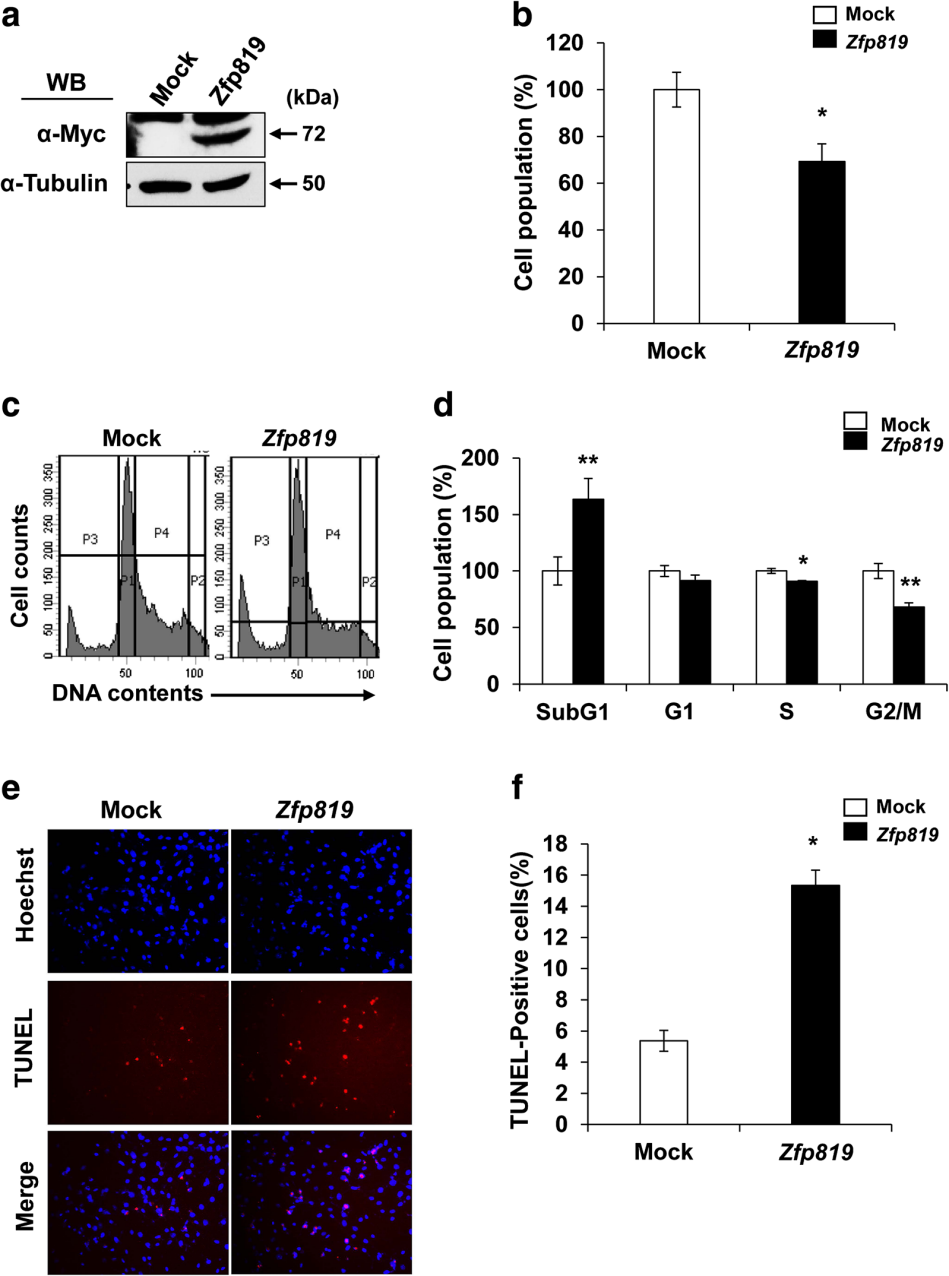
Behavior and apoptosis of *Zfp819*-overexpressing cells. Cells transfected with mock vector or *Zfp819* were analyzed after 48 h. **a** Western blotting of GC-2 cells at 48 h after transfection with *Zfp819* or empty vector. **b** Proliferation of *Zfp819*-overexpressing GC-2 cell line, as assessed by MTT assays. Experiments were repeated three times. The data are expressed as the mean ± SEM; *p < 0.001 (Student’s *t* test). **c**, **d** Cell cycle distribution of *Zfp819*-overexpressing GC-2 cells stained with propidium iodide and assessed by flow cytometry. Histograms of cell cycle distribution (**c**). All experiments were performed three times. The data are expressed as the mean ± SEM; *p < 0.05 and **p < 0.01 versus mock (Student’s *t* test). **e** TUNEL-positive signals (*arrowheads*) were observed using confocal microscopy after 48 h. **f** Histogram of TUNEL analysis. More than 1000 cells were counted. The experiment was repeated three times. The data are expressed as the mean ± SEM; *p < 0.001 versus mock (Student’s *t* test)

A previous study reported that the overexpression of *Zfp819* can induce apoptosis in somatic cell lines (NIH 3T3 and HEK 293T), thereby inhibiting cell proliferation [5]. To confirm whether the effects of *Zfp819* also occur in germ cells, we transfected pcDNA3.1 or pcDNA3.1-*Zfp819* plasmids into GC-2 cells. After 48 h, we confirmed the expression of *Zfp819* in GC-2 cells by western blotting (Fig. 2a). We used the MTT assay to examine the effect of *Zfp819* overexpression on cell proliferation, and found that cell growth was significantly reduced in *Zfp819*-overexpressing GC-2 cells compared with empty-vector controls after 48 h (30% of control) (Fig. 2b). We also confirmed that the cell growth was not changed in another control, *GFP*-overexpressing GC-2 cells (Additional file 2: Figure S1). To determine whether this inhibition of cell proliferation was caused by blockage of the cell cycle, we used flow cytometry to analyze the effect of *Zfp819* on the cell cycle profile. In *Zfp819*-overexpressing cells, a higher proportion of cells were distributed in the sub-G_1_ phase (163% of control), while lower proportions were distributed in the S and G_2_/M phases (91 and 68% of control, respectively), suggesting that the cells were undergoing death (Fig. 2c, d). The G_1_-phase cell population was unchanged. These changes in cell cycle distribution were observed at 48 h post-transfection (Fig. 2c, d).

We used TUNEL assays to determine whether the alterations in the cell populations and cell cycle distributions in *Zfp819*-overexpressing cells were related to apoptosis, and observed more TUNEL-positive (i.e., apoptotic) signals in *Zfp819*-overexpressing cells than in controls (Fig. 2e). Our quantitative analysis demonstrated that *Zfp819* overexpression increased the number of apoptotic cells by approximately threefold compared with controls (Fig. 2f).

### Microarray and ChIP-chip analyses of *Zfp819*-overexpressing GC-2 cells

**Fig. 3:**
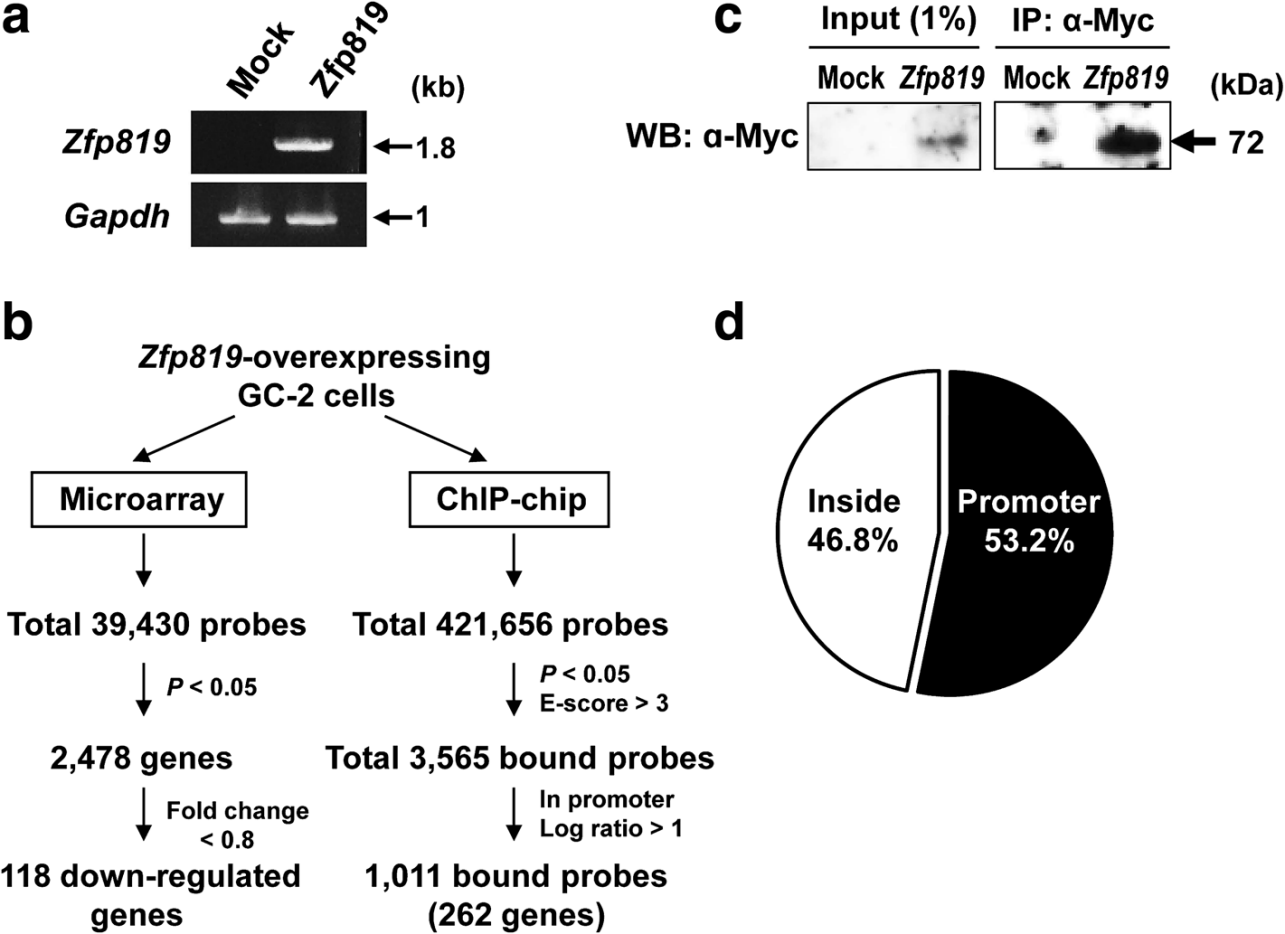
Microarray and ChIP-chip analyses of *Zfp819*-overexpressing GC-2 cells. **a** Overexpression of *Zfp819* in GC-2 cells was confirmed by RT-PCR. The glyceraldehyde-3-phosphate dehydrogenase (*Gapdh*) gene was used as a control. **b** Workflow of microarray and ChIP-chip analyses of *Zfp819*-overexpressing GC-2 cells. **c** Western blotting of immunoprecipitated Zfp819 with α-Myc antibody from GC-2 cells transfected with *Zfp819* or empty vector. **d** Pie chart of chromosomal distribution of *Zfp819* bound peaks

To determine whether *Zfp819* can repress the transcription of endogenous genes and to identify such genes, we performed gene expression profile analysis (microarray) of mock-transfected or *Zfp819*-overexpressing GC-2 cells at 24 h post-transfection. Semi quantitative RT-PCR analysis demonstrated that the *Zfp819* transcripts were not detected in cells transfected with empty vector, but abundantly present in *Zfp819*-transfected cells (Fig. 3a). We also confirmed the expression of *Zfp819* protein in *Zfp819*-transfected cells (data not shown). Microarray analysis of vector- and *Zfp819*-transfected cells using a Mouse GE 8 × 44 K Microarray Kit (Agilent Technology) revealed that a total number of 1737 genes were differentially expressed in *Zfp819*-overexpressing cells compared with controls (*p* value < 0.05) (Fig. 3b). However, out of these genes, only 118 genes were downregulated in GC-2 cells transfected with *Zfp819* when we used a cutoff of a 20% decrease compared with control cells (Additional file 1: Table S2). 11 of the 118 downregulated genes have been previously reported to induce apoptosis when they were silenced (*Cpt1c*, *Ctsh*, *Cbx7*, *Mtdh*, *Sf3b1*, *ELKS*, *Fbxw11*, *Itsn1*, *Rlim*, *Dyrk1b*, and *Rnf121*) (Table 1). Indeed, nine of them showed decreased expression levels in *Zfp819*-transfected cells compared to controls (Additional file 2: Figure S2).

**Table 1:** Down-regulated genes involved in apoptosis by microarray (anti-apoptotic function)

Gene symbol	Description	Gene ontology	Expression level	Ref
Cpt1c	Carnitine palmitoyltransferase 1c	Lipid metabolic process	0.480	[38]
Ctsh	Cathepsin H	Negative regulation of apoptotic process	0.495	[18]
Cbx7	Chromobox 7	Sebaceous gland development	0.509	[20]
Mtdh	Metadherin	Negative regulation of apoptotic process	0.518	[19]
Sf3b1	Splicing factor 3b, subunit 1	Blastocyst formation, RNA splicing	0.590	[39]
Erc1	ELKS/RAB6-interacting/CAST family member 1	Retrograde transport, endosome to Golgi	0.594	[40]
Fbxw11 (HOS)	F-box and WD-40 domain protein 11	Negative regulation of NF-kappaB import into nucleus	0.603	[21]
Itsn1	Intersectin 1 (SH3 domain protein 1A)	Negative regulation of neuron apoptotic process	0.633	[22]
Rlim (Rnf12)	Ring finger protein, LIM domain interacting	Regulation of dosage compensation by inactivation of X chromosome	0.700	[41]
Dyrk1b	Dual-specificity tyrosine-(Y)-phosphorylation regulated kinase 1b	Myoblast fusion	0.705	[42]
Rnf121	Ring finger protein 121	ER-associated ubiquitin-dependent protein catabolic process	0.736	[43]

To further investigate genomic targets regulated by *Zfp819*, we applied ChIP-chip assay using mock or *Zfp819*-overexpressing GC-2 cells. For the purpose of this assay, GC-2 cells were transfected with the *Zfp819* plasmid, and the ChIP assay was performed at 24 h post-transfection. Western blotting confirmed the efficient immunoprecipitation of ZFP819 in *Zfp819*-transfected cells compared with mock-transfected cells (Fig. 3c). Subsequently, the immunoprecipitated DNAs were hybridized on Agilent’s mouse promoter array (400 K), which covers over 400,000 promoter sites, and then analyzed through Agilent’s DNA microarray scanner. As the first step for selecting significant data, we collected probes satisfying two criteria (*p* < 0.05 and E-score > 3) using the Peak Shape Detection V2.1 Model (Fig. 3b). In this process, genes demonstrating low signals on a chip and random selection were removed. As a result, 3565 probes were selected as probes bound by ZFP819 (Fig. 3b). Of these 3565 probes, we found out that the majority of them (53.2%, 1897 probes) were located in promoter regions, whereas 46.8% of the bound probes were located within genes (Fig. 3d). Of the 1897 probes located in promoter regions, we considered those with log ratio (>1) to search for high potential targets of ZFP819. Finally, we identified a total of 1011 probes corresponding to 262 genes as targets bound by ZFP819 in GC-2 cells (*p* < 0.05, E-score > 3, log ratio > 1) (Additional file 1: Table S3, Additional file 2: Figure S3). Binding of ZFP819 to the targets was confirmed in several randomly selected genes by ChIP-PCR (Additional file 2: Figure S4). We further analyzed characteristics of these probes in several ways.

**Fig. 4:**
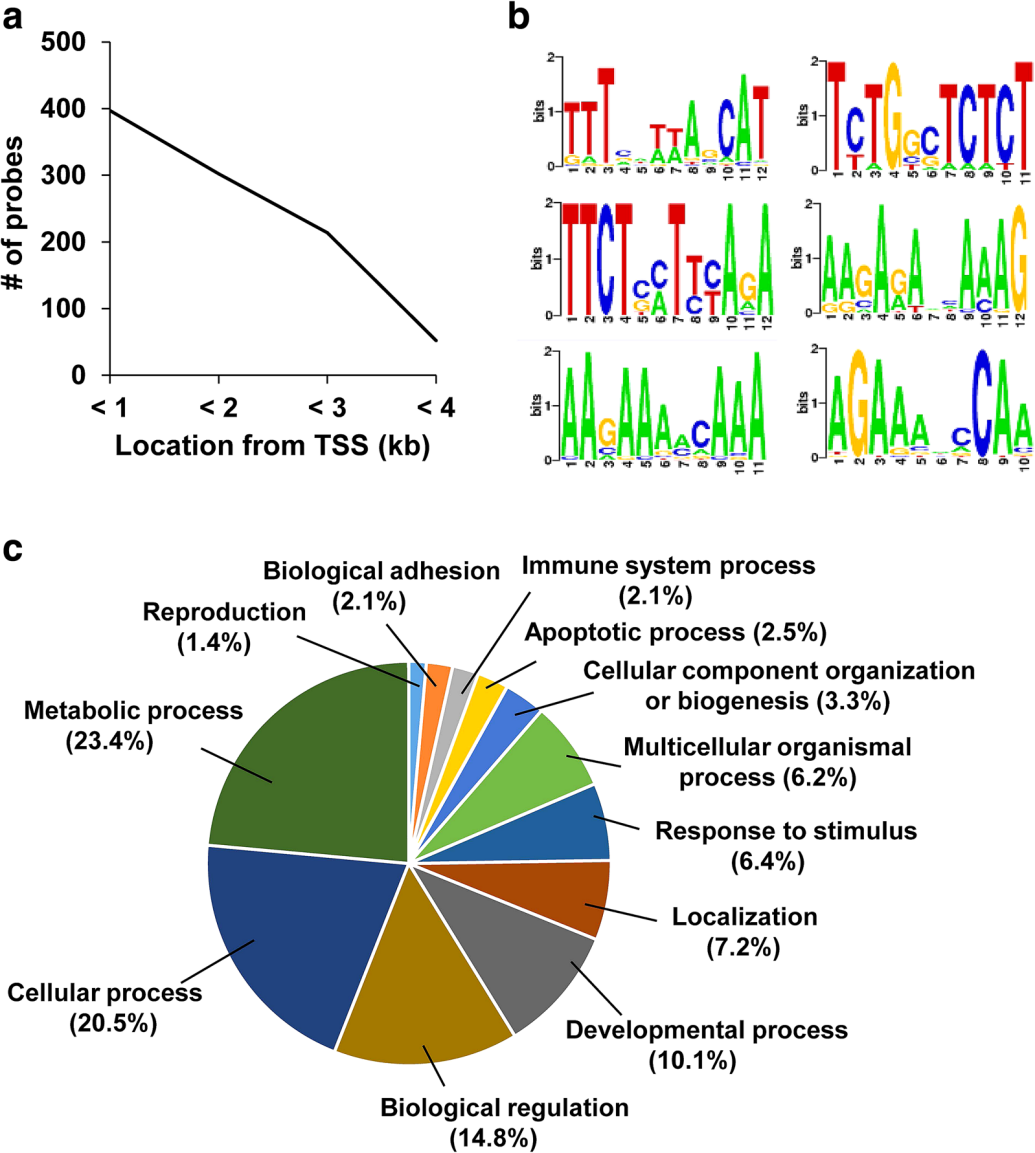
Profiling of ChIP-chip analysis. **a** Location of enriched probes relative to TSS. **b** Enriched sequences of the bound probes. **c** Gene ontology analysis according to a biological process of *Zfp819* target genes from ChIP-chip assay

First, we searched for the location of the ZFP819-bound probes from the transcription start site (TSS) (Fig. 4a). A total of 397 probes (39.2%) among 1011 were located within 1 kb (i.e., proximal promoter region) of the TSS (Fig. 4a). To find out highly represented binding sequences, we analyzed the sequences of 1011 probes using a DMINDA (DNA motif identification and analyses) program (Fig. 4b). The sequence logo according to the position weight matrix demonstrated several sequence motifs were enriched among 1011 probes (1.08e−08 < *p* < 2.89e−06) (Fig. 4b). However, they contain only a small number of probes (9–37 probes). This result indicates that ZFP819 binds to a diversity of sequences rather than consensus elements with a limited number. Gene ontology analysis with a biological process was performed by PANTHER classification system and demonstrated that 262 genes were involved in a variety of processes, such as metabolic, developmental, and apoptosis (Fig. 4c). Finally, we performed a ChIP-PCR analysis several genes were subjected to a ChIP-PCR analysis.

### Identification of genes directly regulated by ZFP819

**Fig. 5:**
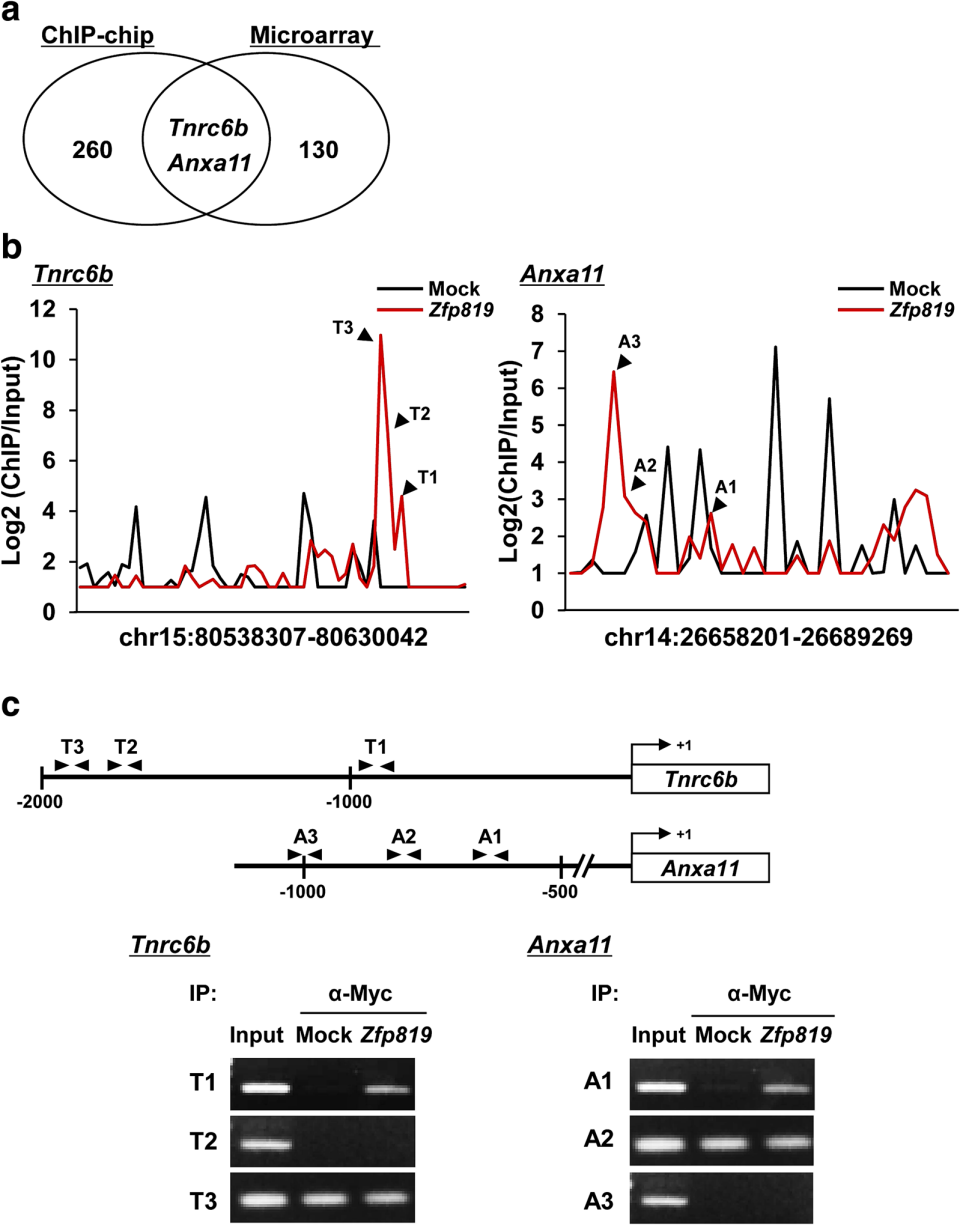
Validation of ChIP-chip analyses. **a** *Venn diagram* representing comparison of the data between microarray and ChIP-chip in *Zfp819*-overexpressing GC-2 cells. **b** *Peak graphs* of *Tnrc6b* or *Anxa11* based on log2 ratio from ChIP-chip assay. *Dotted lines* mean log2 ratio of *Tnrc6b* or *Anxa11* probes from mock-transfected cells. *Black lines* represent log2 ratio of *Tnrc6b* or *Anxa11* probes from *Zfp819*-transfected cells. **c** Schematic structures of *Tnrc6b* and *Anxa11* promoter regions including probes. ChIP-PCR assay was performed with primer sets according to each probe sites (T1–T3 and A1–A3). Input was used as a control

To identify genes directly regulated by ZFP819, we compared the data between microarray and ChIP-chip assay (Fig. 5a). In total, 262 genes gathered from ChIP-chip assay were compared with 118 obtained from microarray analysis, and only two genes that encoded TNRC6B and ANXA11 were commonly found in both of the data (Fig. 5a; Table 2). To test whether ZFP819 binds to the regulatory regions of these genes, we performed a ChIP-PCR assay. To do this, we searched for the enriched regions of the two genes according to Log2 ratio. Peak graphs of *Tnrc6b* and *Anxa11* from a ChIP-chip assay indicated enriched regions in *Zfp819*-overexpressing cells compared with mock cells (Fig. 5b). Considering the distance from the TSS, we performed a ChIP-PCR assay with immunoprecipitated DNAs (Fig. 5c). As demonstrated in schematic structures, a ChIP-PCR assay was processed with primers flanking the promoter sites of *Tnrc6b* or *Anxa11* (T1, −958 to −899; T2, −1815 to −1756; T3, −1950 to −1891; A1, −686 to −626; A2, −834 to −775; A3, −1015 to −956) (Fig. 5c). Of three regions in the *Tnrc6b* promoter, only one site (T1, −958 to −899) was determined to be a region bound by ZFP819. *Anxa11* also revealed that one region (A1, −686 to −626) was amplified in *Zfp819*-overexpressing cells (Fig. 5c).

**Table 2:** Direct targets of ZFP819 by ChIP-chip and microarray

Gene symbol	Description	Gene ontology	ChIP-chip (log ratio)	Microarray (expression level)
Tnrc6b	Trinucleotide repeat containing 6b	Gene silencing by RNA, Regulation of translation	3.455	0.32
Anxa11	Annexin A11	Response to calcium ion	2.688	0.47

**Fig. 6:**
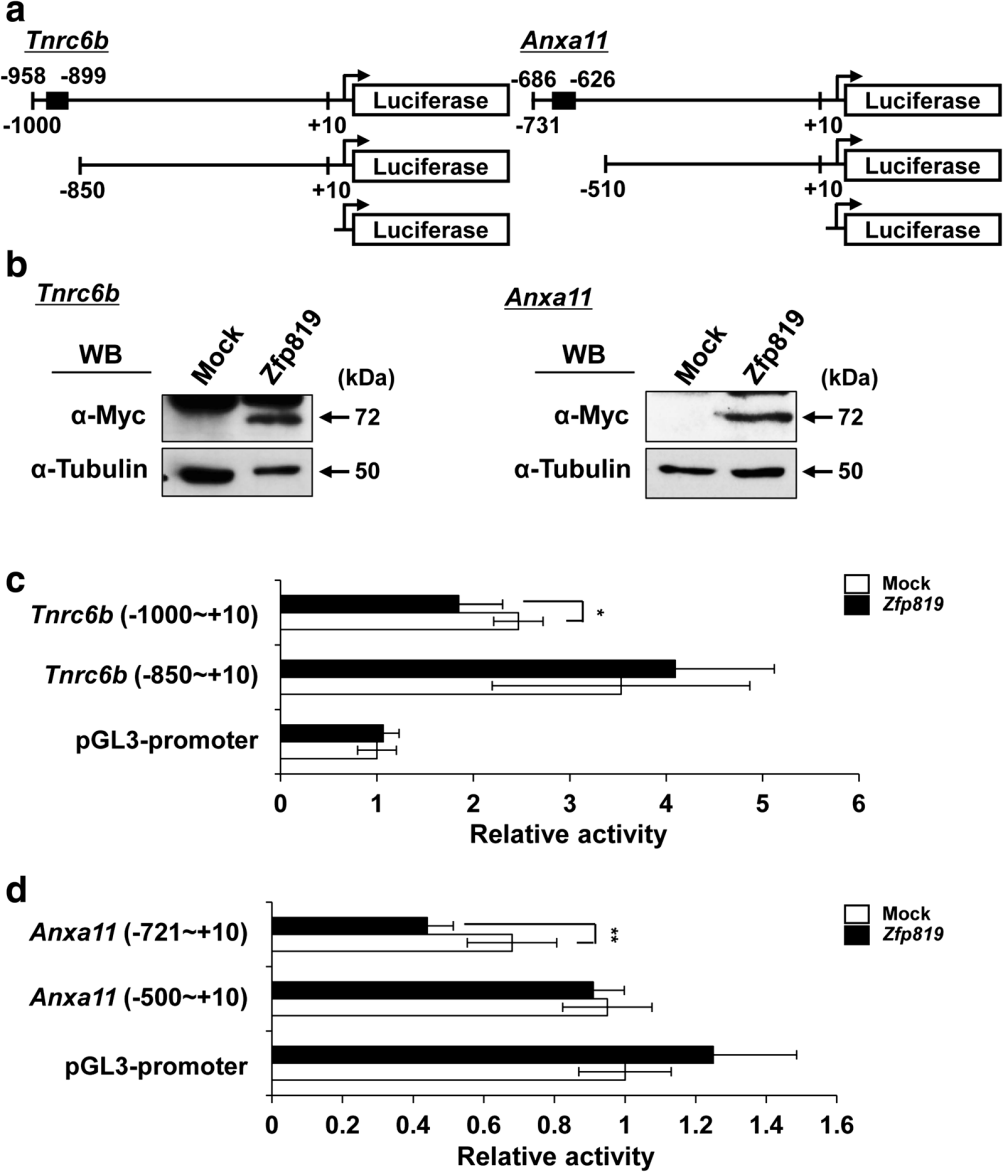
Direct regulation of *Tnrc6b* and *Anxa11* by ZFP819. **a** Schematic diagrams of *Tnrc6b* and *Anxa11* promoters. *Black boxes* mean sites including probes used for ChIP-chip experiment. **b** Western blotting of GC-2 cells at 24 h after transfection with *Zfp819* or empty vector. The α-tubulin protein was used as a control. Analysis of *Tnrc6b* (**c**) and *Anxa11* (**d**) promoters. GC-2 cells were cotransfected with each promoter-luciferase constructs and *Zfp819* or empty vector. Promoter activity was evaluated in dual-luciferase assays. pGL3-promoter vector was used as a control. Experiments were conducted in triplicate. The data are expressed as the mean ± SEM; *p < 0.05, **p < 0.01 versus mock (Student’s *t* test)

Furthermore, we performed a promoter assay according to the binding sites in *Tnrc6b* and *Anxa11* promoters. To this end, we generated luciferase expression constructs with or without the binding sites of *Tnrc6b* and *Anxa11* (Fig. 6a). They were cotransfected with pcDNA3.1 or pcDNA3.1-*Zfp819* plasmids into GC-2 cells. Western blotting demonstrated the stable expression of *Zfp819* protein in GC-2 cells in all of the tests (Fig. 6b). Subsequently, a promoter assay using a dual-luciferase kit was performed at 24 h post-transfection and luciferase activity was measured. The luciferase activity was significantly reduced in −958 to −899 of the *Tnrc6b* promoter in the presence of ZFP819 (Fig. 6c). On the other hand, luciferase activity was not changed when a luciferase expression construct that did not include the site of *Tnrc6b* promoter (−850 to +10) was co-transfected with *Zfp819* or mock (Fig. 6c). We also found that ZFP819 inhibited the luciferase activity driven by −686 to −626 of the *Anxa11* promoter compared with controls, but not the activity driven by a region without −510 to +10 of the *Anxa11* promoter (Fig. 6d). These results demonstrated that ZFP819 directly bound to −958 to −899 in the *Tnrc6b* promoter and to −686 to −626 in the *Anxa11* promoter to regulate gene expression.

### Expression of *Tnrc6b* and *Anxa11* in *Zfp819*-overexpressing GC2-cells

**Fig. 7:**
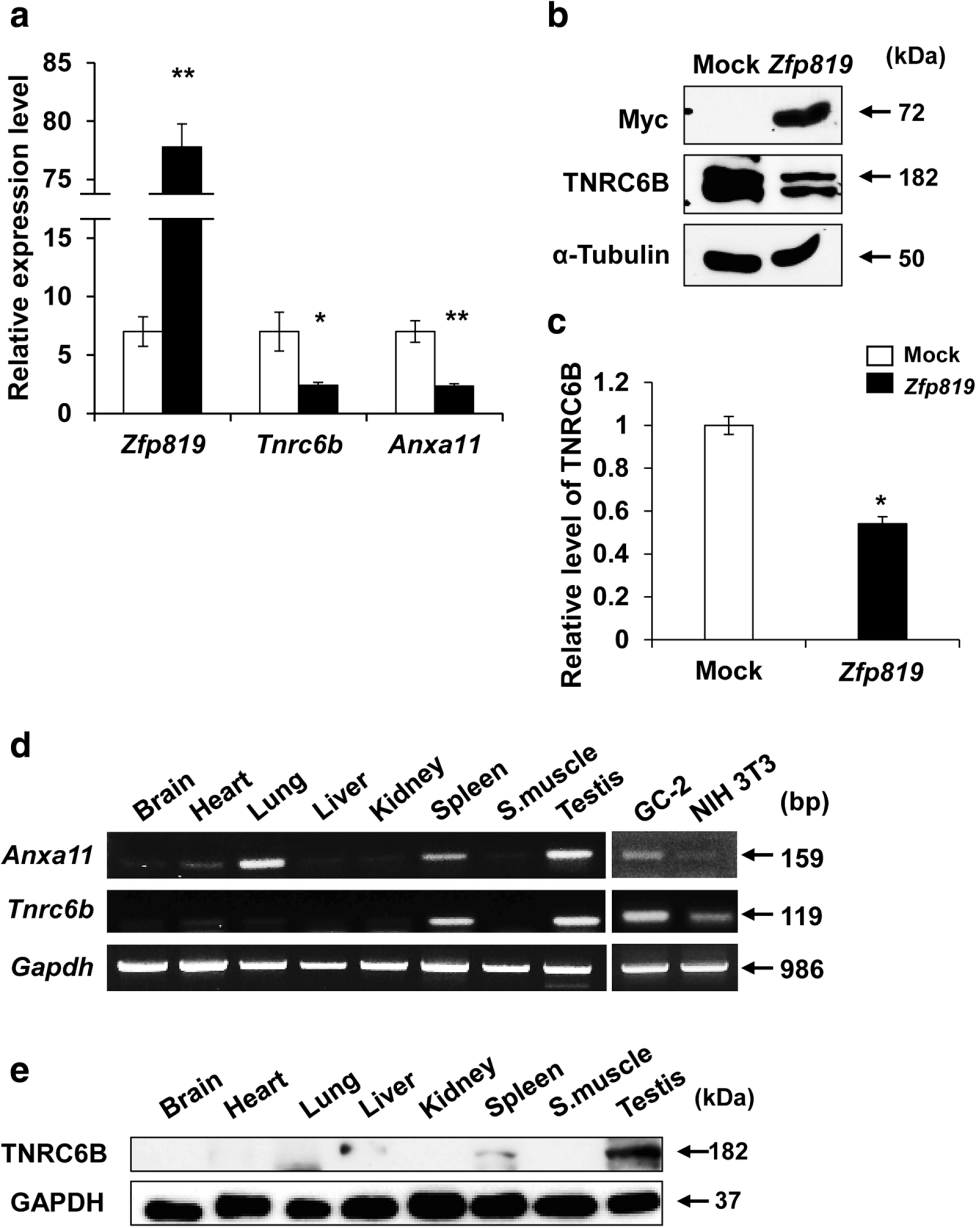
Expression analysis of *Tnrc6b* and *Anxa11*. **a** The transcript levels of *Tnrc6b* and *Anxa11* were examined by qRT-PCR in mock vector- or *Zfp819*-transfected cells. The qRT-PCR data of *Tnrc6b* and *Anxa11* were normalized with that of *Gapdh*. Experiments were repeated three times. The data are expressed as the mean ± SEM; *p < 0.05 and **p < 0.001 (Student’s *t* test). **b**, **c** The expression of TNRC6B in *Zfp819*-overexpressing cells. Expression level of TNRC6B were examined at 48 h post-transfection by Western blotting (**b**), and the intensities of the bands were quantified (**c**). The data are expressed as the mean ± SEM (n = 3); *p < 0.01. **d** Tissue distributions of *Tnrc6b* and *Anxa11*. Complementary DNAs from eight different mouse adult tissues and two different cell lines were subjected to RT-PCR analysis. The glyceraldehyde-3-phosphate dehydrogenase (*Gapdh*) gene was used as a control. **e** Western blotting demonstrated the expression of TNRC6B in eight different mouse adult tissues. GAPDH was used as a control

Then, we examined whether *Zfp819* indeed affects transcription of *Tnrc6b* and *Anxa11* through direct binding to their promoter regions. In agreement with results from promoter assays, quantitative RT-PCR confirmed that both of the transcript levels of two genes were dramatically decreased in *Zfp819*-transfected cells compared with mock cells (Fig. 7a). Moreover, we assessed change in the expression of TNRC6B at the protein level using an available antibody (Fig. 7b). Western blotting confirmed a significant reduction of TNRC6B when *Zfp819* was overexpressed in GC-2 cells (Fig. 7b). Our quantitative analysis demonstrated that *Zfp819* overexpression decreased the expression of TNRC6B by approximately 50% of control levels (Fig. 7c). However, we could not test the expression of *Anxa11* because of the unavailability of an antibody that works in Western blot analysis. Until now, the expression patterns of *Tnrc6b* and *Anxa11* have not been investigated in various tissues. Thus, we examined the tissue distributions of *Tnrc6b* and *Anxa11* through in vitro expression analysis (Fig. 7d). Interestingly, *Tnrc6b* and *Anxa11* were predominantly expressed in testis and GC-2 cells (Fig. 7d). Moreover, Western blotting also confirmed the predominant expression of TNRC6B in testis (Fig. 7e). Finally, we investigated the effects of siRNA-mediated gene silencing of *Tnrc6b* and *Anxa11* in GC-2 cells. The knockdown efficiency was confirmed by RT-PCR. This analysis showed that the cell proliferation was significantly inhibited in *Tnrc6b*- or *Anxa11*-knockdown GC-2 cells (Tnrc6b, 54% of control; Anxa11, 67% of control), consistent with the phenotype observed in *Zfp819*-transfected cells (Additional file 2: Figure S5).

## Discussion

Here, we investigated the transcriptional network influenced by *Zfp819* in a germ cell line. We previously identified the downstream genes of *Zfp819* in NIH 3T3 cells by overexpression-microarray analysis [5]. Because of some advantages, such as low cytotoxicity, minimal optimization, and high transfection efficiency, we used the NIH 3T3 cell line in the previous study. However, we needed to assess the transcriptional mechanism of *Zfp819* in germ cells due to the specific expression of *Zfp819*. GC-2 cells, originally from mouse spermatocytes, have features in common with morphological differentiation at the early spermatid stages [11]. However, these features were not observed in another germ cell line, GC-1 cells derived from spermatogonia. Thus, the GC-2 cell line is an essential tool to study cellular effects that occur in spermatocytes. The functions of several transcription factors have been studied in GC-2 cells [12, 13]. On the basis of these facts, we investigated the genomic targets of *Zfp819* using GC-2 cells, thereby looking into the actual downstream genes of *Zfp819* in a system as close to germ cells as possible. It should be noted that most of the differentially expressed genes were found to be upregulated in the microarray analysis. We do not know how the overexpression of *Zfp819* leads to the larger number of upregulated genes, considering that ZFP819 is a transcriptional repressor. Perhaps, some of these genes were indirectly upregulated by the downregulated genes. Alternatively, the upregulated genes could be the result of compensatory response of cells undergoing changes by the overexpression of *Zfp819.* We focused on the downregulated genes to identify and investigate genes directly regulated by ZFP819.

A comparison between the data of microarrays revealed that there were hardly overlapped genes in two cell lines (NIH 3T3 and GC-2) [5], even though *Zfp819* overexpression showed the same phenotype (induction of apoptosis). This suggests that *Zfp819* regulates different upstream genes in different pathways, eventually involved in apoptosis. Intriguingly, however, the different genes of TNRC6 family showed down-regulation in *Zfp819*-overexpressing cells, which are *Tnrc6b* and *Tnrc6a* in GC-2 and NIH 3T3 cells, respectively. The members of TNRC6 family (also GW182, glycine-tryptophan repeats) are well known as the major components in miRNAs-mediated gene silencing [14]. For the functions, they interact with Argonautes (AGOs) which is the central effectors [14, 15]. The TNRC6 members have the functional redundancy [16], and thus, been speculated about playing a role in a tissue-or cell-specific manner [17]. We suppose that *Zfp819* regulates gene expression of members in TNRC6 family, each different members in different cell lines.

Of 118 down-regulated genes by microarray, we found 10 genes showing an anti-apoptotic function in the previous studies (Table 1). Two (*Ctsh*, and *Mtdh)* of them regulate expression of proteins belonging to Bcl-2 family [18, 19]. *Cbx7* as a component of polycom repressive complex 1 has functions in cancer cell development and extension of cellular life span [20]. A recent paper demonstrated that apoptotic cells increased via expression of tumor necrosis factor-related-apoptosis-inducing ligand (TRAIL) in *Cbx7*-silenced condition [20]. *Fbxw11* and *Itsn1* are involved in apoptosis by modulating expression of downstream genes in mitogen-activated protein kinases (MAPK) family [21, 22]. On the other hand, seven genes selected by microarray in NIH 3T3 cells play a role in apoptosis by affecting the expression of downstream effectors of p53 or depending on p53 [5, 23–25]. Thus, the selected genes in both cell lines control the expression of multiple steps in apoptosis, ultimately activating Caspase-3 or PARP, although they individually regulate several proteins in different pathways.

Furthermore, we defined the genomic target sites of *Zfp819* by ChIP-chip technique in GC-2 cells. Of the enriched 1011 promoter sites (262 genes) by *Zfp819*, surprisingly, only two genes (*Tnrc6b* and *Anxa11*) were selected by two different genome-wide approaches (microarray and ChIP-chip). This suggests that most of the down-regulated genes in the microarray analysis are indirectly regulated by *Zfp819.* Alternatively, it is possible that transfection efficiency of *Zfp819* influences on expression of downstream genes, in spite of the same condition in two different experiments. In addition, ChIP-chip technique sometimes generates a high ratio of signal-to-noise, thereby selecting a large number of peaks [26]. These results were also obtained in other similar studies [27–29].

So far, apoptosis has been considered an important process at every stages during spermatogenesis [30]. As undergoing mitosis, meiosis, and post-meiosis sequentially, cells need to keep a balance between proliferation and apoptosis for producing normal sperm. There is high possibility of occurring many of errors for mitosis and meiosis, and cell death by apoptosis is necessary to remove those cells with generic defects. In terminal differentiation, apoptosis generally occurs to remove cytoplasmic components, producing highly specialized cells.

Both *Tnrc6b* and *Anxa11* are heavily involved in apoptosis. As mentioned the above, TNRC6B is an interacting partner of Ago2 and forms a complex with miRNA induced-silencing complex (miRISC) [15, 31]. This complex participates in two different ways for silencing of mRNA: mRNA decay and translational repression [32]. In these processes, miRNAs have an important role by recognizing their mRNA targets. MiRNAs are essential to control the temporal and spatial gene expression at every stages during spermatogenesis as well [33]. For example, miR-34c, expressed from pachytene spermatocyte, promotes the apoptosis of germ cells via balancing expressions between the *Bcl*-*2* and BCL2 associated X protein (*Bax*) genes [34]. Additionally, miR-34c activates the expression of transcription factor 1 (*Atf1*), and then decreases *Bax* expression in knockdown of *Atf1*, consequently, inducing germ cell apoptosis [34].

*Anxa11* encodes a calcium-dependent phospholipid-binding protein. During cell cycle, ANXA11 shows a dynamic pattern from nucleus to nuclear envelop with co-localization of S100A6, a small calcium binding protein [35, 36]. In *Anxa11*-silenced condition, the siRNA-transfected cells appeared the incompletion of cytokinesis generating many of binucleate cells, thereby leading to apoptosis by increasing the expression of PARP [36]. So far, the mechanism that *Anxa11* is involved in apoptosis exactly has not been discovered. A recent study indicated that the knockdown of *Anxa11* decreased proliferation and survival of hepatocarcinoma cells by increasing expression of thymoma viral proto-oncogene 2 (AKT2) and phospholylated forkhead box O1 (FOXO1) [37]. Thus, this suggests that Anxa11 is involved in cell survival or apoptosis in different cell types.

## Conclusion

In summary, we herein investigate cellular effects and target genes influenced by *Zfp819* in GC-2 cells. The overexpression of *Zfp819* induced apoptosis and inhibited cellular proliferation. We identified two genes regulated by *Zfp819* by microarray and ChIP-chip analysis. Interestingly, both of two genes showed the predominant expression in testis. Previously, these genes were found to play an important role in apoptosis. Taken together, our study provides new information about germ cell-specific gene regulation by *Zfp819* which potentially functions in maintaining the integrity of spermatogenesis through apoptosis.

## Additional files

**Additional file 1: Table S1.** List of primers used in this study.** Table S2.** Downregulated genes by ZFP819 using microarray analysis.** Table S3.** Promoter sites bound by ZFP819 using ChIP-chip analysis.

**Additional file 2: Figure S1.** Cell proliferation of Zfp819-overexpressing cells.** Figure S2.** Expression of nine down-regulated genes in Zfp819-overexpressing cells.** Figure S3.** Chromosomal map of peaks bound by ZFP819.** Figure S4.** ChIP-PCR assay.** Figure S5.** Knockdown effects of Tnrc6b and Anxa11 in GC-2 cells.
