# Supplementary material for: Identification of target genes for spermatogenic cell-specific KRAB transcription factor ZFP819 in a male germ cell line
Source: Cell Biosci. 2017 Jan 3;7:4. doi: 10.1186/s13578-016-0132-4 (PMC5209904; doi:10.1186/s13578-016-0132-4)
Supplement: Supplementary file 2 — Additional file 2: Figure S1. Cell proliferation of Zfp819-overexpressing cells. Figure S2. Expression of nine down-regulated genes in Zfp819-overexpressing cells. Figure S3. Chromosomal map of peaks bound by ZFP819. Figure S4. ChIP-PCR assay. Figure S5. Knockdown effects of Tnrc6b and Anxa11 in GC-2 cells. [file 13578_2016_132_MOESM2_ESM.pdf]

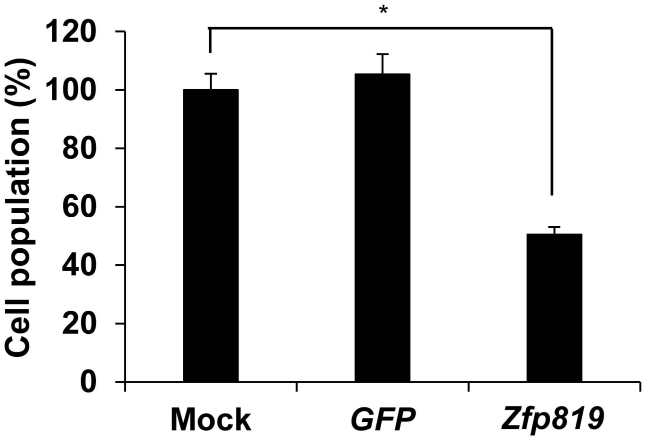

Figure S1. Cell proliferation of *Zfp819*-overexpressing cells. Cells transfected with mock, *GFP* or *Zfp819* were analyzed after 48 h. Cell population was assessed by MTT assays. Experiments were repeated three times. The data are expressed as the mean ± SEM; *$p < 0.01$ (Student's t-test).

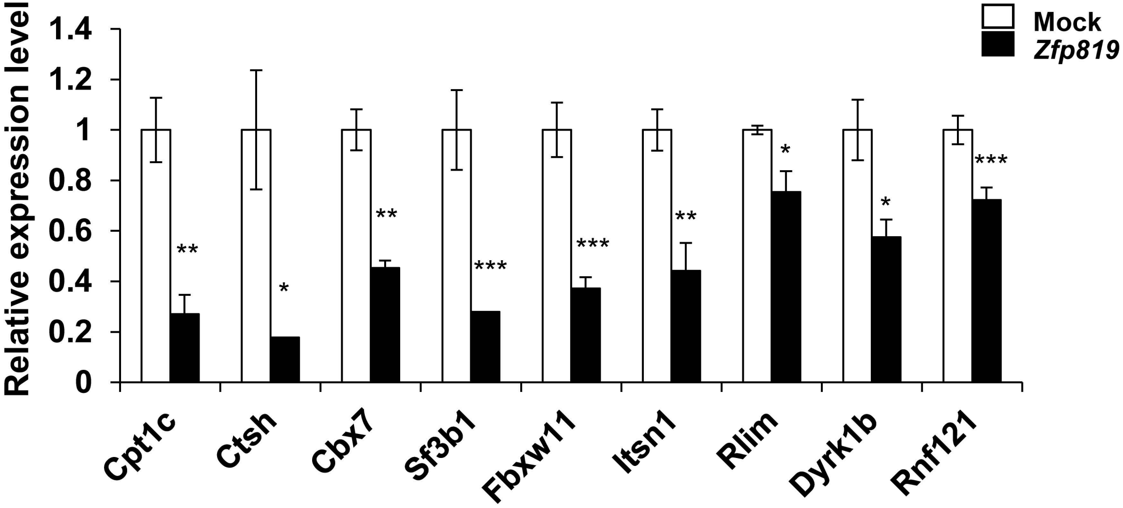

Figure S2. Expression of nine down-regulated genes in *Zfp819*-overexpressing cells. qRT-PCR was performed in mock- or *Zfp819*-transfected cells. Experiments were repeated three times. The data are expressed as the mean ± SEM; *$p < 0.05$, **$p < 0.01$, ***$p < 0.001$ (Student's t-test).

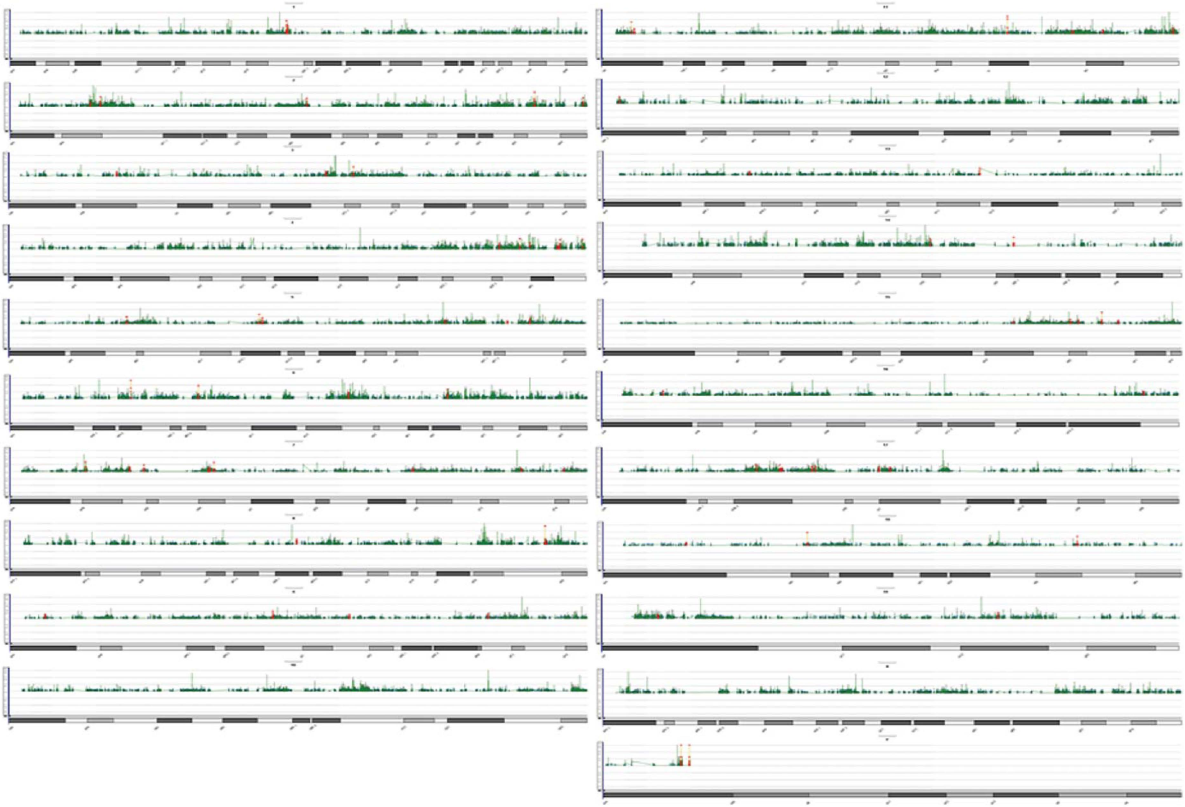

Figure S3. Chromosomal map of peaks bound by ZFP819.
Red dots demonstrate enriched sites. Green peaks represent background signals.

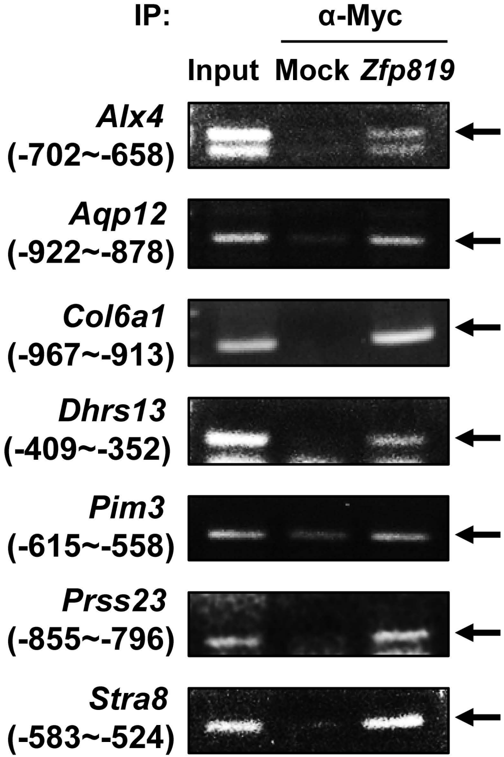

Figure S4. ChIP-PCR assay. Genes randomly selected according to log ratio were subjected to ChIP-PCR with gene specific-primer sets amplifying the ZFP819-binding sites (shown in parentheses). Input was used as a control.

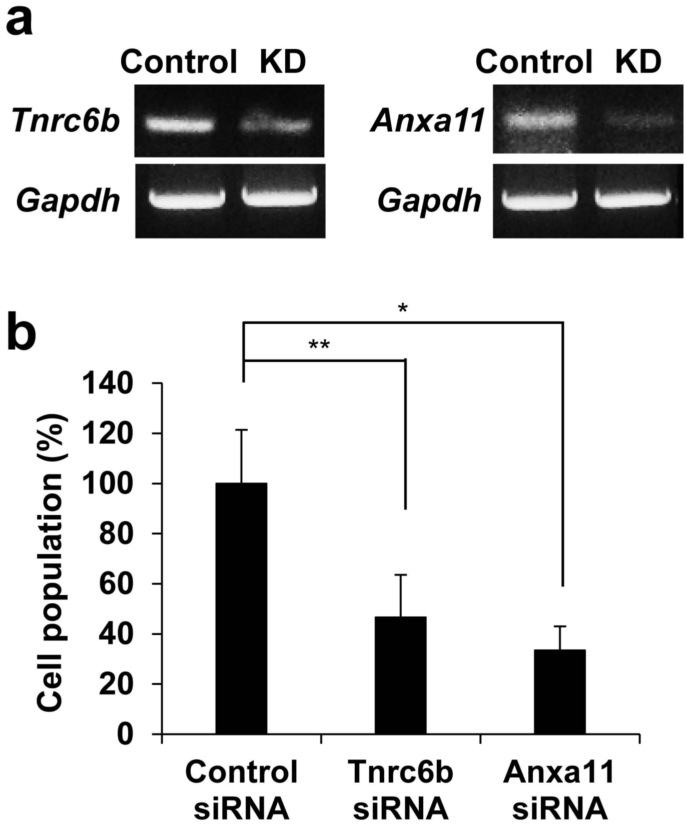

Figure S5. Knockdown effects of Tnrc6b and Anxa11 in GC-2 cells. **a** Cells transfected with control siRNA, Tnrc6b siRNA or Anxa11 siRNA were analyzed after 48 h. **b** Cell population was assessed by MTT assays. Experiments were repeated three times. KD, knockdown. The data are expressed as the mean ± SEM; *$p < 0.05$, **$p < 0.001$ (Student's *t*-test).
